# High-Quality Cellulosic Fibers Engineered from Cotton–Elastane Textile Waste

**DOI:** 10.1021/acs.biomac.3c01366

**Published:** 2024-02-22

**Authors:** Lorena Villar, Inge Schlapp-Hackl, Pablo B. Sánchez, Michael Hummel

**Affiliations:** † Department of Chemical Engineering, University of Vigo, Vigo 36310, Spain; ‡ Department of Bioproducts and Biosystems, School of Chemical Engineering, Aalto University, Espoo 02150, Finland

## Abstract

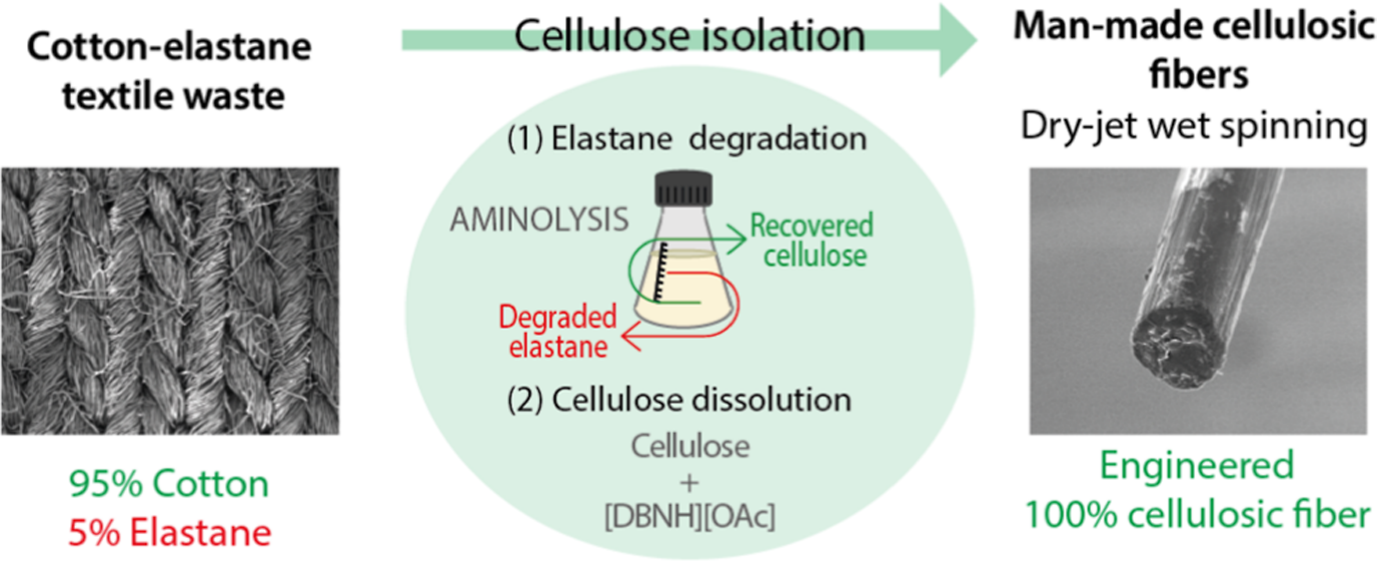

Even small amounts of elastane in cotton–elastane blended textiles can prevent fiber-to-fiber recycling strategies in textile recycling. Herein, the selective separation of elastane from cotton blends was addressed by the aminolytic degradation of the synthetic component. Polar aprotic solvents were tested as elastane solvents, but side reactions impeded aminolysis with some of them. Aminolysis of elastane succeeded under mild conditions using dimethyl sulfoxide in combination with diethylenetriamine and 1,5-diazabicyclo[4.3.0]non-5-ene as a cleaving agent and catalyst, respectively. The analysis of the nitrogen content in the recovered cellulose fraction demonstrated that 2 h of reaction at 80 °C reduced the elastane content to values lower than 0.08%. The characterization of the recovered cellulose showed that the applied conditions did not affect the macromolecular properties of cellulose and maintained a cellulose I crystal structure. Degraded elastane products were recovered through precipitation with water. Finally, the cellulosic component was turned into new fibers by dry-jet wet spinning with excellent tensile properties.

## Introduction

Globally, 53 million tons of fibers are consumed annually for clothing purposes, but less than 1% are recycled into new clothes.^1^ Cellulose-based fibers dominate staple fiber production, accounting for 64% in 2021.^2^ Among all of the benefits of cellulose as a natural fiber, textile blends of cellulose with synthetic materials aim to improve the specific properties of the fabrics. Textile recycling can be achieved by mechanical or chemical treatments, depending on the quality and composition of the residues. Mechanical recycling is a down-cycling process since the quality of the fibers is reduced through shredding or cutting steps.^1,3^ Via chemical recycling, a true recycling and, in some cases, even upcycling process is possible by the dissolution and regeneration of the polymer. Chemical recycling of cellulose into high-quality man-made cellulosic fibers (MMCFs) via Ioncell, a Lyocell-type dry-jet wet spinning technique, was already studied.^4^ This technique enables fiber-to-fiber recycling by the conversion of textile waste into second-generation fibers of high quality. The regenerated fibers often exceed the properties of the initial materials.^5,6^

**Figure 1:**
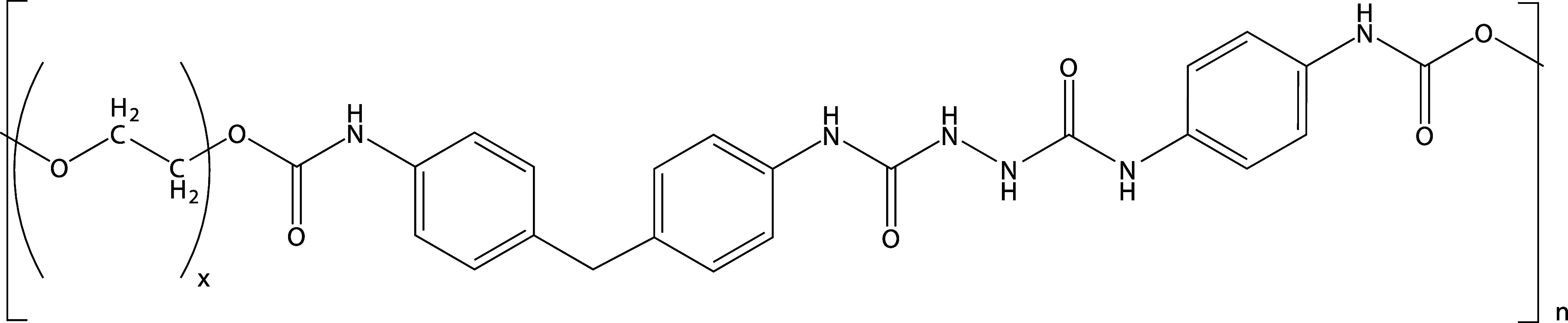
Structure of the repeating unit of elastane.

Chemical recycling becomes a challenge when textile blends are involved. Polyester and elastane are examples of synthetic fibers commonly blended with cellulose. Most synthetic fibers are prepared via melt spinning of petrochemical-based polymers. However, natural fibers cannot be melt-processed, and solution spinning is needed for the recycling of natural fibers.^7^ These differences in filament production hamper a common recycling route. The incorporation of elastane provides a significant stretch to the cotton fabric, allowing it to expand and recover its original shape when stretched. This property makes the fabric more flexible, comfortable, and accommodating to body movements.^8^ Adding low amounts (between 2 and 5%) is enough to improve the function and comfort of cotton fabrics.^9^ Cotton–elastane blends can be found as core-spun yarns, where the synthetic filament is located in the core of the yarn and surrounded helically by the main component,^10^ or as plated plain knitted fabrics, where both components are merged in a complex matrix.^11^ Recently, the separation of elastane from other synthetic fiber blends was addressed by selective dissolution using tetrahydrofurfuryl alcohol, a green alternative to classical solvents.^12^ In particular, thermal degradation routes have been widely studied. Robello et al. developed a method to separate and recycle a polyamide–elastane blend under controlled conditions of temperature, pressure, and humidity.^13^ The separation of elastane from nylon was also addressed by the selective degradation of elastane via heat treatment.^14^ The thermal degradation of elastane fibers involves the use of high temperatures, around 200 °C, without affecting the polyamide component of the yarn. However, these harsh conditions cannot be applied to cellulose-based blends since the high temperatures likely induce cellulose degradation.^15^ Chemical degradation methods are an alternative to selectively separate elastane from cellulose without damaging the natural fiber. Designing a process to degrade the elastomeric phase requires a thorough understanding of the elastane structure (Figure 1). This polymer is composed of two segments linked by strong covalent urethane bonds. The rigid segment is formed by aromatic isocyanates, which are responsible for intermolecular hydrogen bonds, giving strength and stability to the material. It is the so-called hard segment and represents the crystalline domain. The second segment is flexible, also known as the soft segment, which contains polyols such as polyether and creates amorphous domains that provide elasticity.^16,17^ The polymer chains are linked by intermolecular hydrogen bonds that form a strong matrix that is characterized by relatively high chemical stability.^18^

Chemical depolymerization of polyurethane was previously studied by several methods such as acidolysis or glycolysis using high temperatures.^19−22^ Focusing on the urethane bonds present in the elastane structure, aminolysis can be a suitable alternative to common depolymerization treatments due to the mild conditions involved, preventing thermal damage of the cellulosic component.^23^ In the aminolysis of elastane, the urethane bonds are attacked by an amine through nucleophilic addition, followed by the elimination of the alkoxy group. As a result, polymer chains are split into two segments. Polyols and substituted amines are the products of the depolymerization reaction.^24^ Among the amines that were studied as cleaving agents of polyurethanes,^25,26^ diethylenetriamine (DETA) was described as the most convenient depolymerization reactant since it improved the reaction rate and reduced the viscosity of the collected products in solution.^27^ In addition, the depolymerization rate was directly related to the basicity of the degrading agent.^25^ Recently, Van Dam and co-workers patented a method for the separation of elastane from cellulosic fibers.^28^ In that research, they stated the use of a solvent system containing dimethylformamide (DMF) as an elastane solvent, DETA as a cleaving agent, and different catalysts that shorten the reaction time. Up to 99.5% of elastane was removed, and the polymerization degree of the remaining cotton was not significantly affected. However, environmental concerns can be associated with the use of some organic solvents, such as DMF.^29^

This work focuses on the isolation of the cellulosic component in cotton–elastane blends, which is typically the main fraction (95% or higher). Elastane was removed through aminolytic degradation to yield a pure cellulose substrate that was dry-jet wet spun into MMCFs. More precisely, elastane was degraded by aminolysis using DETA as a cleaving agent. Several polar aprotic solvents were tested as a greener alternative to DMF: γ-valerolactone (GVL), dihydrolevoglucosenone (Cyrene), tetrahydrofuran (THF), and dimethyl sulfoxide (DMSO). After aminolysis, the cellulosic component was studied to assess the efficiency of the separation and to ensure that the applied conditions did not damage the natural polymer. Finally, the cellulosic material was dissolved and turned into new fibers for textile applications via dry-jet wet spinning.

## Materials and Methods

### Materials

A white preconsumer textile blend, composed of 95% (w/w) of cotton and 5% (w/w) of elastane, was purchased from Eurokangas (Finland). The fabric was ground using a Wiley mill coupled to a 30 μm sieve and dried (dry-matter content ≥98%) before being used as the starting material (SM). DETA (C_4_H_13_N_3_, *M* = 103.17 g/mol, CAS: 111-40-0, purity 99.0%), DMF (C_3_H_7_NO, *M* = 73.09 g/mol, CAS: 68-12-2, purity ≥99.8%), DMSO (C_2_H_6_OS, *M* = 78.13 g/mol, CAS: 67-68-5, purity 99.5), and Cyrene (CAS: 53716-82-8, C_6_H_8_O_3_, *M* = 128.13 g/mol, purity ≥ 98.5%) were supplied by Merck. 1,5-Diazabicyclo[4.3.0]non-5-ene (DBN, C_7_H_12_N_2_, *M* = 124.18 g/mol, CAS: 3001-72-7, purity 99.0%) was provided by Fluorochem. GVL (C_5_H_8_O_2_, *M* = 100.12 g/mol, CAS: 108-29-2) was produced in the laboratory following published procedures.^30^ All solvents were used without further purification. Cellulose dissolution was carried out using 1,5-diaza-bicyclo[4.3.0]non-5-enium acetate ([DBNH][OAc]) synthesized in the laboratory following the procedure explained in preceding works.^31^

### Elastane Degradation by Aminolysis

A similar procedure to Van Dam and co-workers was carried out for the aminolytic degradation of elastane.^28^ 8 g of the ground fabric was added to 160 mL of a solvent mixture containing a cleaving agent (DETA) and an elastane solvent (DMF or DMSO) in a 1:1 volume ratio. Additionally, 100 μL of DBN was added as a catalyst. The mixture was mechanically stirred for 4 h at 80 °C. Then, the solid and liquid phases were separated by filtration. The cotton that remained in the solid phase was washed first with the respective solvent and then with distilled water. The liquid phase contained the solvent mixture and the degraded elastane products. Finally, adding water to the liquid phase caused the precipitation of the elastane products.

### Analysis of the Recovered Materials

The nitrogen and carbon contents of the recovered materials were quantified using a Fisons Carlo Erba microanalyzer. Thermal behavior was studied by thermogravimetric analysis (TGA) using a NETZSCH STA 449 in a dynamic mode from 50 to 650 °C with a heating ramp of 10 °C min^–1^ under He flow (70 mL min^–1^). Fourier transform infrared spectroscopy (FTIR) measurements were performed on a PerkinElmer spectrometer in the wavelength range 400–4000 cm^–1^. Fourier self-deconvolution (FSD) was applied to optimize FTIR results in the range of 3000–3700 cm^–1^, with a bandwidth of 100 cm^–1^ and an enhancement of 2°. Liquid ^1^H and ^13^C NMR spectra were recorded at 400 MHz and 64 scans using a Bruker DPX 400 device.

### Recycling of Cellulosic Fibers

Intrinsic viscosity ([η]) of cellulose was calculated from the kinematic viscosity in a cupriethylenediamine (CED)/water mixture, following the standard SCAN CM 15:88.^32^ Cellulose was depolymerized by acid hydrolysis (H_2_SO_4_ 0.05 M at 80 °C) until reaching values of [η] between 400 and 600 mL g^–1^. The dope was prepared with a cellulose concentration of 13% (w/w) via the dissolution of the polymer in [DBNH][OAc] at 85 °C and 30 rpm for 150 min under reduced pressure (around 50 mbar), followed by high-pressure filtration (200 bar) to remove insoluble particles. Viscoelastic properties of the resulting solution were recorded with an Anton Paar MCR 302 rheometer (gap size: 1 mm, dynamic mode) equipped with a 25 mm plate within angular frequency and temperature ranges of 0.01–100 rad s^–1^ and 50–90 °C, respectively. Complex viscosity (η*) and dynamic moduli (*G*′, *G*″) were evaluated as a function of the angular frequency (ω), and zero shear viscosity (η_0_^*^) was determined under the assumption of the validity of the Cox–Merz rule.^33^ The solution was spun by dry-jet wet spinning. In this process, the dope was gradually extruded through a single-hole spinneret (diameter: 0.1 mm, length-to-diameter ratio: 0.2) with a velocity of 1.3 m min^–1^ at 70 °C. The dope was spun into an aqueous coagulation bath (5 °C) passing through an air gap of 5 mm that allowed for drawing the liquid filament. Fibers were collected at draw ratios (DR = *V*_take-up_/*V*_extrusion_) of 5, 8, and 11.

### Evaluation of the Collected MMCFs

Changes in the molar mass distribution (MMD) of the cellulose throughout the process were determined by gel permeation chromatography (GPC) with an Agilent 1260 Infinity HPLC, following published methods.^34^ The separation system consisted of three PLgel 20 μ m MIXED-A 300 mm × 7.5 mm Agilent columns connected in series (mobile phase: 0.5% LiCl/DMAc; injection volume: 100 μ L). The flow rate was 0.5 mL·min^–1^, and each sample run took up to 70 min. All samples were filtered through a 0.45 μm poly(tetrafluoroethylene) (PTFE) syringe filter. Ten pullulan standards with nominal masses ranging from 800 kDa to 320 Da provided by Fluka were used for calibration. Wide-angle X-ray scattering (WAXS) was performed by the use of a Xenocs Xeuss 3.0 (detector: 2D Dectris Eiger2 R 1M; sample-to-detector distance: 56 mm) with CuK_α_ radiation in transmission mode operated with 50 kV and 0.6 mA at a wavelength of 1.54189 Å. The initial cotton–elastane sample and the recovered cotton sample were grinded and fixed as a powder to the sample holder via Kapton tape. The cotton fibers were positioned as a bundle unidirectionally, vertically to the X-ray beam and to the sample holder without any tape. The scattering patterns of each sample were collected at three different positions. A blank measurement was performed without any sample from the air and with Kapton tape. The sample profiles were corrected by the background scattering profiles via subtraction of the intensities. The Segal crystallinity for the cellulose I and II patterns was determined according to Nam et al.^35^ The tensile properties of the spun fibers (20 fibers per sample) were measured using a TexTechno Favigraph fiber tester with a gauge length of 20 mm and a testing speed of 20 mm·min^–1^, equipped with a 20 cN load cell and pretension weights of 150 mg (DR5) and 100 mg (DR8 and DR11), in both conditioned (20 °C, 65% humidity) and wet (10 s wetting before being tested) states. Fiber morphology was analyzed by scanning electron microscopy (SEM) using a JEOL JSM-6700f microscope.

## Results and Discussion

### Solvent Selection for Aminolysis

**Figure 2:**
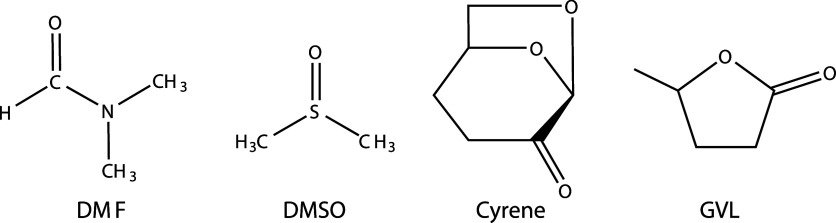
Structure of the solvents used in this work.

In aminolysis, the urethane group is attacked by the amine (herein DETA) acting as a nucleophile, causing the formation of alcohol and amine-segments (Figure S1 shows the hypothesized mechanism of the degradation route^24^). Besides, an elastane solvent is added to the system with the aim of promoting fragmentation as well as the dissolution of the products of the reaction. DMF has been reported in literature as an elastane solvent for aminolysis, but it is connected to several environmental concerns.^28^ In an effort to find alternative solvents with minor associated risks, the following solvents were tested: DMSO, Cyrene, and GVL (Figure 2). All three solvents are polar and aprotic, which could potentially lead to a solvent behavior similar to DMF.^29^ In a recent research study authored by Phan et al.,^12^ these solvents were proposed among a broad screening of suitable solvents for the selective dissolution of elastane based on Hansen solubility (HS) parameters and COSMO-RS predictions. However, aminolytic degradation was a requisite to induce elastane dissolution under the conditions studied in this work.

HS parameters are widely used to evaluate polymer–solvent affinity. These parameters are described by the energy from the individual contribution of three interactions: dispersion (δ_*d*_), polar (δ_*p*_), and hydrogen bond (δ_*h*_).^36^ Comparable parameters between solvents imply similar behavior when interacting with a solute. Generally, solvents with high δ_*h*_ values are preferred to promote the disruption of the hydrogen bond network and facilitate dissolution.^37^ Despite slight differences in their polar contribution, DMF and DMSO had similar HS values with a high δ_*h*_ parameter (Table 1). However, Cyrene and GVL showed noticeable differences; their low δ_*h*_ values suggest a different solvent behavior that may also affect their ability to dissolve elastane. Apart from HS data, Kamlet–Taft (KT) solvatochromic parameters determine the ability of a solvent to interact as a hydrogen bond donor (α), hydrogen bond acceptor (β), or its polarizability (π*) capacity.^38^ Similar KT parameters were reported for all of the solvents involved in this work (Table 1). All of them had high β and π* values, as was expected based on their molecular structure.

**Table 1:** HS and KT Parameters of the Solvents Involved in This Work

		DMF^39^	DMSO^39^	Cyrene^39^	GVL^40,41^
HS (MPa^1/2^)	δ_*d*_	17.4	18.4	18.9	15.5
	δ_*p*_	13.7	16.4	12.4	4.7
	δ_*h*_	11.3	10.2	7.1	6.6
KT	α	0	0	0	0
	β	0.69	0.76	0.61	0.60
	π*	0.88	1	0.93	0.83

Changes in solution properties were experimentally observed when some of the solvents were added to the reaction mixture. In the case of Cyrene, a highly viscous liquid was formed after an exothermic reaction with DETA. Also, the viscosity of the mixture increased when GVL was added to the system. Although these solvents were recently proposed as green elastane solvents by Phan et al., these changes in solution properties evidenced that their reactivity with amine compounds can be a limitation for their use in the aminolytic degradation of elastane.^12^ Above 50 °C, Cyrene was reported to be unstable in basic media as it undergoes aldol condensation.^42,43^ Moreover, Chalid and co-workers proposed a mechanism for the ring-opening reaction of GVL with amino compounds, suggesting that the nucleophilicity of the amines is a decisive factor.^44^ Therefore, Cyrene and GVL had to be discarded as solvents for elastane under the conditions used in this work. The viability of DMF and DMSO as elastane solvents is similar.

### Aminolytic Degradation and Analysis of the Recovered Products

**Figure 3:**
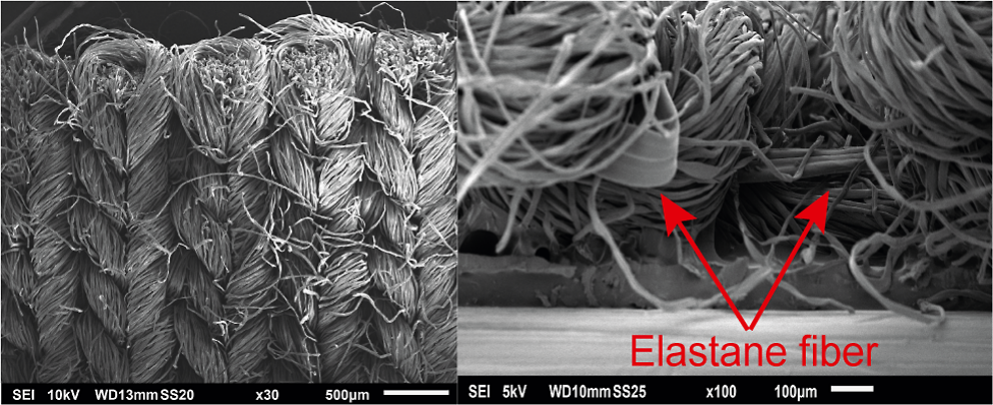
SEM images of the surface (left) and cross-section (right) of the SM (composition: 95% cellulose, 5% elastane).

Elastane degradation was carried out under mild conditions to preserve the cellulosic material. SEM images from the surface and cross-section of the SM are shown in Figure 3.

**Figure 4:**
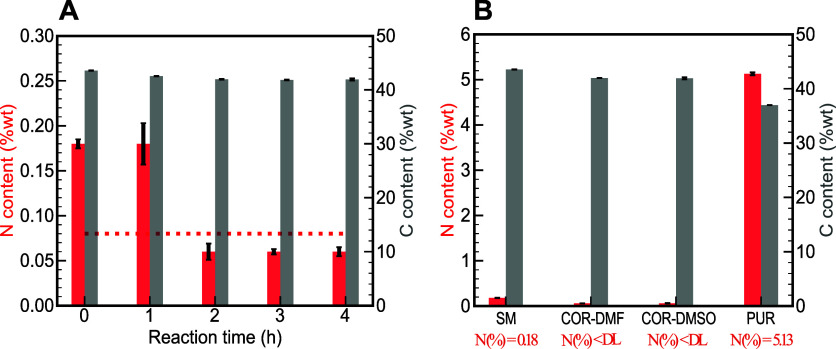
(a) Evaluation of the nitrogen and carbon content of the cellulosic component throughout the aminolysis reaction. The red dotted line indicates the detection limit for nitrogen content (0.08%). (b) Comparison of the nitrogen and carbon content of the SM, the recovered cellulosic component (COR) using both DMF and DMSO as solvents, and the recovered product from polyurethane degradation (PUR). All measurements were performed in triplicate, and the error bars in the graph depict the standard deviation.

The lower temperature limit for the aminolysis reaction was found to be 80 °C.^24^ The optimization of the reaction conditions is key to avoiding uncontrolled cellulose degradation. For this purpose, the reaction time was optimized by isolating and washing small fractions of the solid component every hour during aminolysis using DMSO as a solvent until reaching a maximum of 4 h. The samples were analyzed by elemental analysis to assess the separation of elastane through the decrease in nitrogen content in the cellulosic component. Results are plotted in Figure 4a. The nitrogen content of the SM was notably reduced after 2 h of aminolysis, reaching values lower than the detection limit (DL) of the analyzer. Hence, DMSO is a suitable alternative solvent to DMF for the aminolytic degradation of elastane, reducing the environmental impact of the procedure.

**Figure 5:**
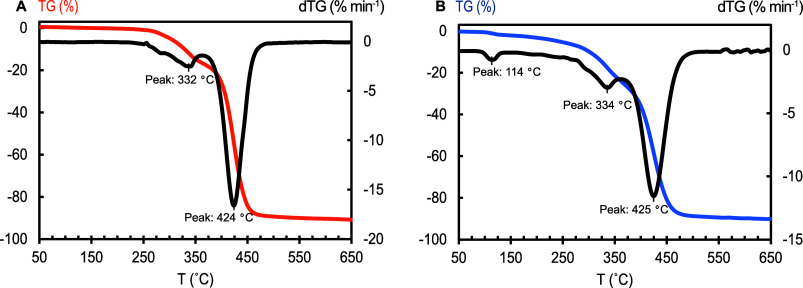
Mass loss (%) and derivative weight (%/min) within a temperature range of 50–650 °C for Es (a) and PUR (b) samples.

The nitrogen content of the SM and the recovered cellulose (COR) under the optimized conditions using DMF and DMSO solvents (COR-DMF and COR-DMSO, respectively) is depicted in Figure 4b. The results confirmed the feasibility of DMF and DMSO as elastane solvents. Regarding the liquid phase, degraded components from elastane that remained soluble in the solvent system after aminolysis were recovered by precipitation upon addition of water. The recovered material from polyurethane degradation products (PUR) showed a high nitrogen content. The precipitate was further analyzed to evaluate its composition. TGA was previously applied to confirm the presence of the two segments from polyurethanes, giving rise to a two-stage degradation profile.^45^ The weight loss of an elastane sample (Es) isolated from the SM as well as the recovered product (PUR) was recorded as a function of temperature. The mass loss (TG) and the first derivative (dTG) curves of both samples are shown in Figure 5. Both samples showed similar thermal behavior. Two degradation steps were observed in the Es sample (Figure 5a) that were attributed to the hard and soft segments of elastane. The presence of two stage degradation in the PUR sample (Figure 5b) at a similar temperature to Es (first stage degradation at 334 °C and second stage at 425 °C) confirmed the presence of the two segments in the PUR. It is noted that the weight loss at 114 °C is due to the presence of water from the recovery process. Although the presence of both segments in the PUR sample was indicated by the TGA results, the thermal behavior does not allow for any conclusions concerning the connectivity between those segments.

FTIR and NMR analyses of PUR were performed to collect information on its chemical structure. The FTIR spectra of Es and PUR (Figure S2) showed the characteristic peaks of polyurethane; only slight differences on low-intensity peaks that are not specific of polyurethane compounds were observed. The reduced solubility of Es and PUR in common organic solvents hampered the analysis of the samples by ^1^H and ^13^C NMR. Figures S3 and S4 show the ^1^H NMR and ^13^C NMR spectra of the studied materials in DMF-d6. The signals of the solvent can be clearly distinguished; the additional peak in the PUR sample may be due to the presence of some solvent used in aminolysis. The low resolution of the peaks corresponding to the materials impeded any multidimensional analysis of H–H and C–H bondings. However, adding water to the system induced the precipitation of both segments. This can explain the presence of the signals corresponding to both segments, despite the absence of a covalent linkage between them.

**Figure 6:**
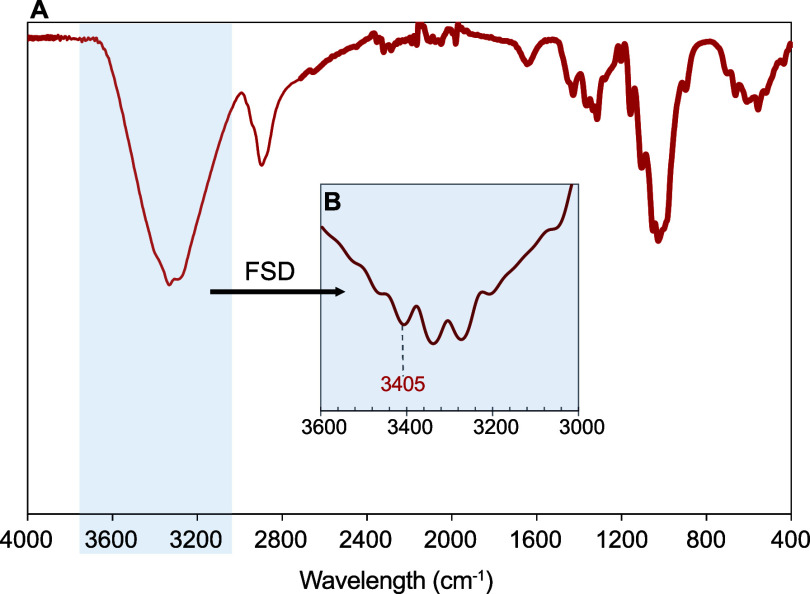
(a) FTIR spectra of COR using DMSO as a solvent. (b) FSD of the spectra in the range 3000–3600 cm^–1^. The specific peak of the cellulose I structure is indicated.

The effect of the reaction on COR materials was determined by FTIR to discard any possible damage to the polymer of interest. The FTIR spectra of COR, shown in Figure 6a, exhibited the expected signals for cellulosic materials. In addition, possible changes in the intermolecular arrangement of the COR were studied in the range from 3000 to 3600 cm^–1^, where the irreversible evolution of cellulose I to cellulose II could be exhibited.^46^ FSD was applied in the region to reveal overlapping spectral features that contribute to the absorption bands (Figure 6b). The specific peak of the crystalline cellulose I structure was found at 3405 cm^–1^ in the spectrum of COR, indicating that the crystalline domains were not affected during the aminolytic treatment of elastane. These results are in agreement with those found for other fibers studied in the literature.^46^

### Recycling of COR into MMCFs

In the following, only cellulose recovered after aminolysis using DMSO was converted into virgin Lyocell-type fibers.

In Lyocell spinning, such as in the Ioncell process, a certain degree of polymerization (DP) of the cellulosic substrate is necessary to obtain solutions with the viscoelastic properties for dry-jet spinning.^47^ Measurements of the intrinsic viscosity of the COR showed high DP values (around 3000), confirming that the cellulosic fraction was not degraded under the selected experimental conditions. Acid treatment was applied to reduce the intrinsic viscosity of the polymer within the recommended range that is considered adequate for spinning ([η] = 400–500 mL·g^–1^).^48^ The spinning dope was prepared via the dissolution of the treated cellulose in [DBNH][OAc], and the rheological properties of the dope were measured to identify suitable spinning conditions. A zero shear viscosity (η_0_^*^) close to 30 000 Pa·s, a crossover point (COP) at around 1 s^–1^, and a modulus between 4000 and 5000 Pa were found to be suitable for cellulose (13% eucalyptus pulp) spinning in [DBNH][OAc].^5^ In the case of the prepared dope, no COP was observed within the frequency range measured (Figure S5). The zero shear viscosity (η_0_^*^) of the spinning solution (14% total polymer concentration) was 170,000 Pa·s at 70 °C. This difference can be explained by the relatively high molecular weight of the cellulose even after acid hydrolysis ([η]_COR_ = 581 mL·g^–1^). This value is close to the upper limit for dopes with good spinnability.^5^ Fibers were regenerated by dry-jet wet spinning. The dope was gradually extruded through a single-hole spinneret, and fibers were collected at different DRs by changing the speed of the godets onto which the filaments were collected.

Changes in the cellulose crystallinity throughout the process were evaluated through WAXS analyses. The X-ray diffraction profiles of the SM and COR samples illustrated a cellulose I pattern (1–10:14.9, 110:16.4, 200:22.6, 004:34.6°) and the regenerated cotton fibers a cellulose II pattern (1–10:12.7, 110:20.3, 020:22.0, 004:34.9°) (Figure S6a).^49^ Thereby, a Segal crystallinity of 72 ± 2% (SM) and 70 ± 1% (COR) was calculated for the cellulose I patterns, respectively. The regenerated CO fibers reached a Segal crystallinity of 69 ± 1%. These crystallinities are in the range of the results achieved by Nam et al. (cellulose I cotton: 84.7%, cellulose II cotton: 68.8%),^35^ indicating that the treatment does not cause any changes in the ratio of the amorphous-crystalline fractions. The evolution of MMD of the cellulosic component was recorded by GPC (Figure S6b). The similar molecular weight between the SM and the COR (corresponding to DP values of 4980 and 3572, respectively) confirmed that aminolysis conditions did not significantly affect the natural polymer. However, acid treatment and, to a lesser extent, dissolution-regeneration steps lowered the DP of the recycled fibers to 1970.

### Evaluation of the Collected Fibers

Regenerated fibers were collected continuously at DRs of 5, 8, and 11. Moreover, the maximum DR at which the fiber can be drawn before breaking was tested, and the resulting value of DR 16 qualifies the dope quality as excellent for spinning.^50^ The DR is directly connected to the fiber diameter and the orientation of the cellulose chains, having an effect on the tensile properties of the collected fibers.

**Figure 7:**
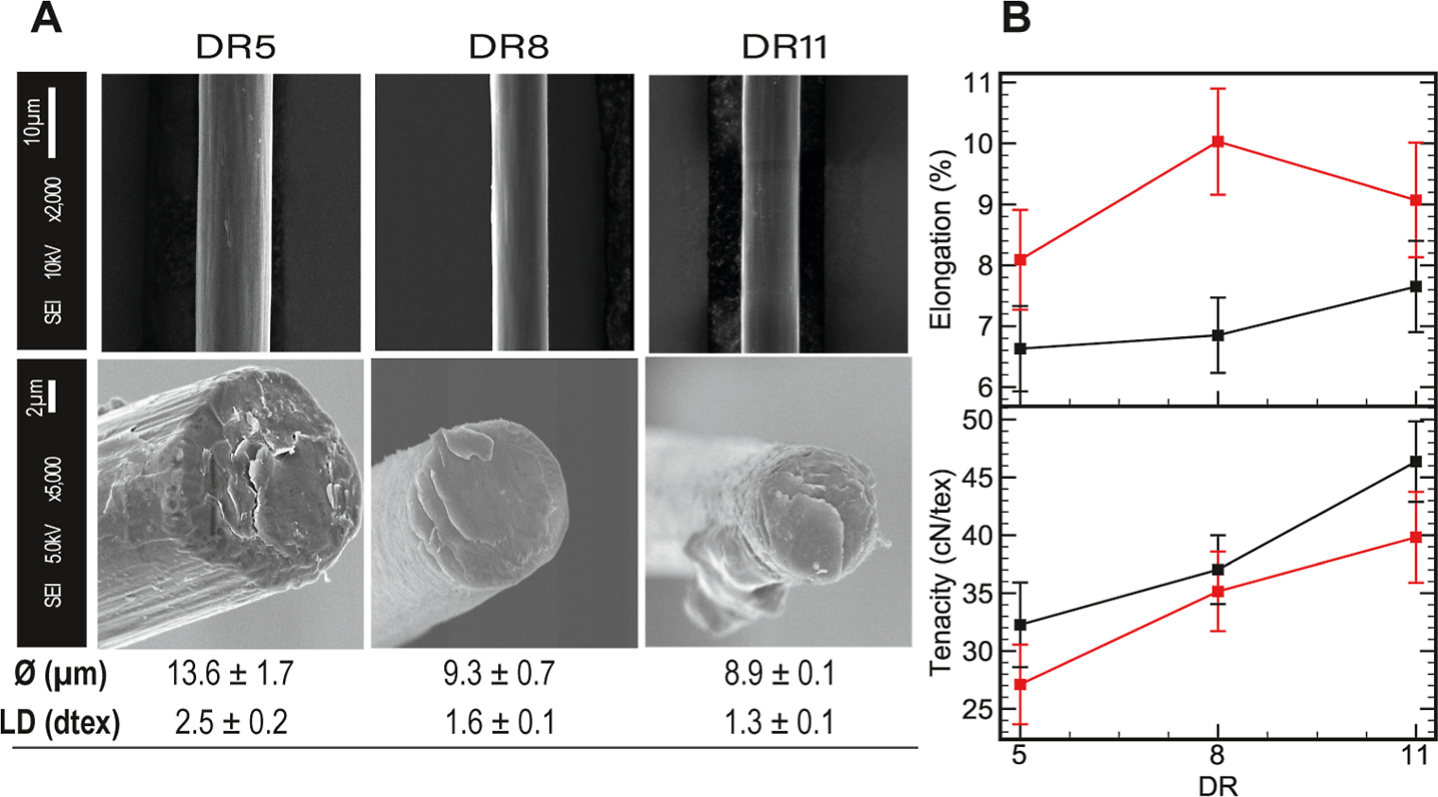
(a) SEM images of the surface and cross-section of collected fibers at different DRs, with diameter and LD values. (b) Elongation and tenacity behavior of the collected fibers as a function of the DR in conditioned (black) and wet (red) states. Tensile tests were repeated 20 times for each fiber; the error bars in the graph depict the standard deviation.

The morphology of the fiber surface and cross-section of filaments spun at different DRs was analyzed by means of SEM, as shown in Figure 7a. All fibers exhibited a smooth surface and a circular cross-section with a fibrillar assembly in the bulk, which is typical of Lyocell-type fibers.

The diameters of the collected fibers were measured from the SEM images and are correlated with the linear density (LD), and the resulting values are shown in Figure 7a. Both parameters decreased as expected: DR5 > DR8 > DR11. In addition, the total orientation and crystallinity of the cellulose chains are improved with higher DRs, which determine the tensile properties of the fiber. The average stress–strain curves of the collected fibers in conditioned and wet states are plotted in Figure S7. Figure 7b shows the influence of the DR on the elongation and tenacity of conditioned and wet fibers. The tenacity values were in the range of previously reported Ioncell cellulosic fibers and surpass most commercial MMCFs currently on the market.^5^ Moderate elongation was also observed earlier when using waste cotton as a cellulose substrate.^48,50^ The increase in elongation and decrease in strength under wet conditions are characteristic of MMCFs and are due to the swelling of amorphous domains. This effect is slightly reduced at high DRs because of the higher orientation of cellulose molecules and their higher crystallinity.^51^

## Conclusions

The recycling of textile blends consisting of cellulosic and synthetic fibers requires a separation step to ensure fiber-to-fiber recycling of cellulose. In this work, aminolysis was presented as a promising option to selectively remove elastane from cellulose in mixed-polymer textiles. GVL, Cyrene, THF, and DMSO have been studied as alternative solvents to DMF for the aminolytic degradation of elastane. Although all of them were regarded as potential alternatives based on their KT and HS parameters, side reactions in the presence of amine impede their use for aminolytic degradation. Only DMSO was identified as a greener alternative to DMF for elastane degradation in the presence of DETA (cleaving agent) and DBN (catalyst) under mild conditions. After 2 h of reaction, the nitrogen content of the cellulosic component was lower than 0.08%. The COR was regenerated into new fibers via dry-jet wet spinning at different DRs, showing the highest mechanical properties at DR11 (46.4 cN/tex tenacity, 7.7% elongation, in the conditioned state). Aminolytic fragments of elastane were precipitated from the liquid phase through the addition of water. Further analysis of these degraded products from polyurethane is needed if we aim for possible revalorization of these elastane fragments.

## Supporting Information Available

Aminolytic degradation mechanism, FTIR, and NMR of PUR; viscoelastic properties of the spinning solution; WAXS profiles and MMD curves of the cellulosic component throughout the process; and stress–strain curves of recycled cellulosic fibers (PDF)
