# Supplementary material for: High-Quality Cellulosic Fibers Engineered from Cotton–Elastane Textile Waste
Source: Biomacromolecules. 2024 Feb 22;25(3):1942–9. doi: 10.1021/acs.biomac.3c01366 (PMC10934812; doi:10.1021/acs.biomac.3c01366)
Supplement: Supplementary file 1 — bm3c01366_si_001.pdf [file bm3c01366_si_001.pdf]

High-quality cellulosic fibers engineered from cotton-elastane textile waste

Lorena Villar,† Inge Schlapp-Hackl,‡ Pablo B. Sánchez,*,† and Michael Hummel*,‡

†*Department of Chemical Engineering, University of Vigo, Vigo 36310, Spain*

‡*Department of Bioproducts and Biosystems, School of Chemical Engineering, Aalto University, Espoo 02150, Finland*

E-mail: pabsanchez@uvigo.es; michael.hummel@aalto.fi

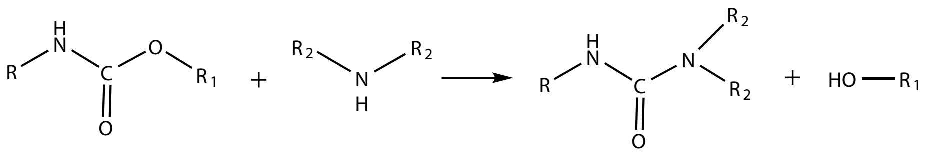

Figure S1: Possible mechanism for the aminolytic degradation of elastane.

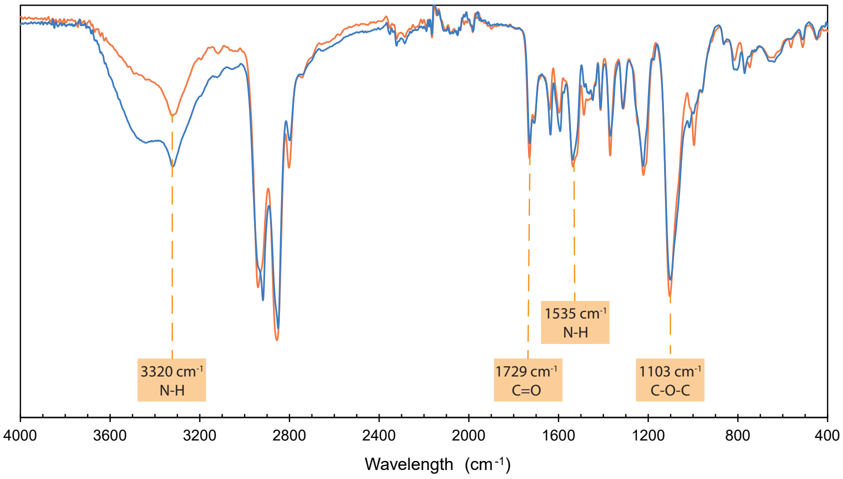

Figure S2: FTIR spectra of elastane (Es-orange) and the recovered product (PUR-blue). The characteristic peaks of elastane are indicated in the figure.

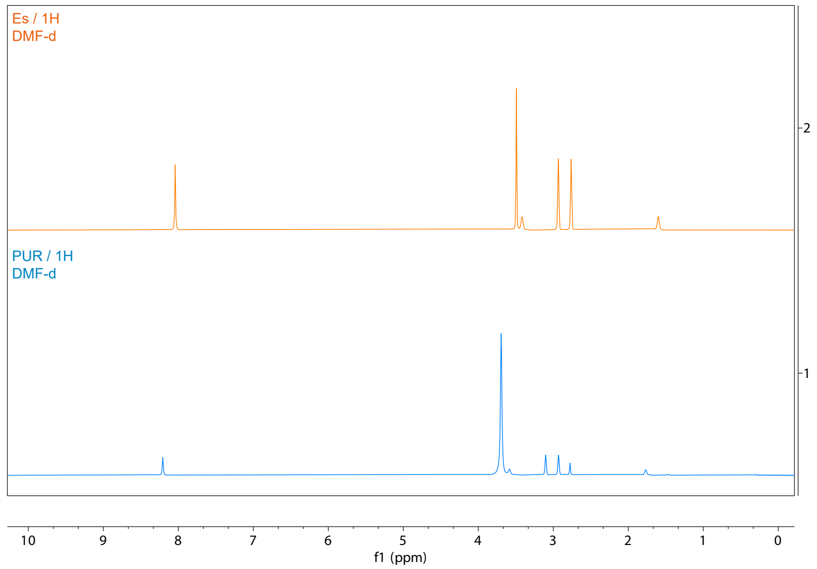

Figure S3: ^{1}H NMR of elastane (Es-orange) and the recovered product (PUR-blue). Both samples dissolved in DMF-6d.

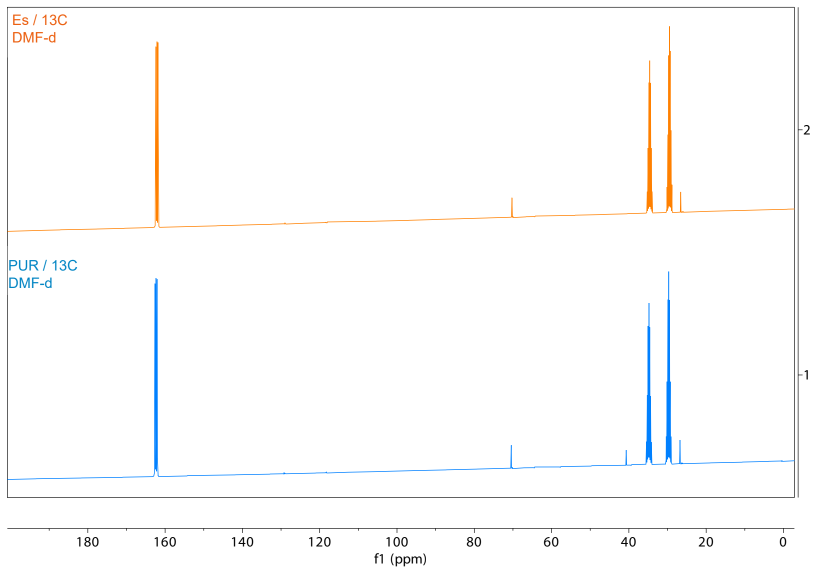

Figure S4: ^{13}C NMR of elastane (Es-orange) and the recovered product (PUR-blue). Both samples dissolved in DMF-6d.

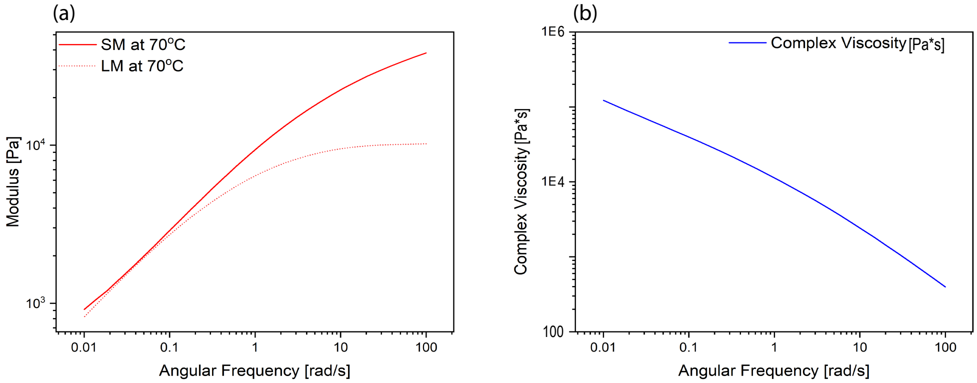

Figure S5: Logarithmic illustration of the viscoelastic properties of the spinning solution at 70 °C presenting storage modulus (SM), loss modulus (LM) vs angular frequency (a) and complex viscosity vs angular frequency (b).

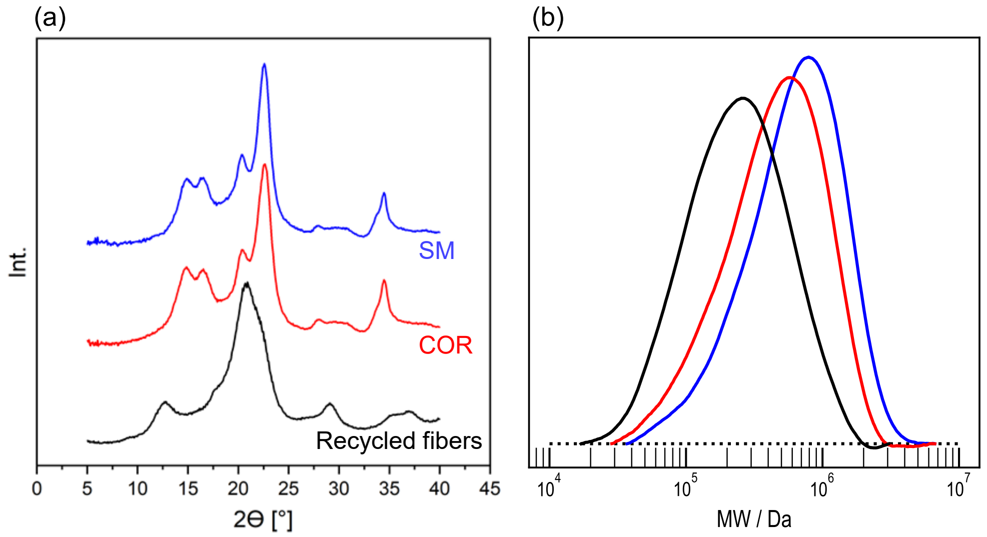

Figure S6: X-ray diffraction profiles (a) and MMD curves (b) of the cellulosic material throughout the process: starting material (blue), recovered cotton (red) and recycled fiber samples (black).

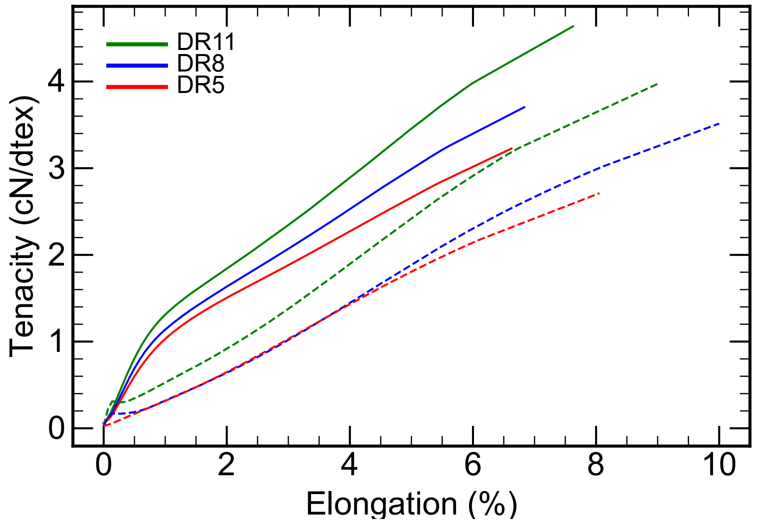

Figure S7: Stress-strain curves of the recycled fibers in conditioned (solid lines) and wet (dotted lines) states. Fibers collected at DR5 (red), DR8 (blue) and DR11 (green).
